# Risk for Acquiring Coronavirus Disease Illness among Emergency Medical Service Personnel Exposed to Aerosol-Generating Procedures

**DOI:** 10.3201/eid2709.210363

**Published:** 2021-09

**Authors:** Aubrey Brown, Leilani Schwarcz, Catherine R. Counts, Leslie M. Barnard, Betty Y. Yang, Jamie M. Emert, Andrew Latimer, Christopher Drucker, John Lynch, Peter J. Kudenchuk, Michael R. Sayre, Thomas Rea

**Affiliations:** University of Washington, Seattle, Washington, USA (A. Brown, C.R. Counts, B.Y. Yang, A. Latimer, J. Lynch, P.J. Kudenchuk, M.R. Sayre, T. Rea);; Public Health Seattle and King County Division of Emergency Medical Services, Seattle (L. Schwarcz, L.M. Barnard, J.M. Emert, C. Drucker)

**Keywords:** aerosol-generating procedures, aerosol transmission, cardiac arrest, coronavirus disease, COVID-19, emergency medical services, emergency treatment, health occupations, medical first responders, public health, public health readiness, respiratory infections, SARS-COV-2, severe acute respiratory syndrome coronavirus 2, viruses, zoonoses

## Abstract

We investigated the risk of coronavirus disease (COVID-19)­ patients transmitting severe acute respiratory syndrome coronavirus 2 (SARS-CoV-2) to emergency medical service (EMS) providers, stratified by aerosol-generating procedures (AGP), in King County, Washington, USA, during February 16–July 31, 2020. We conducted a retrospective cohort investigation using a statewide COVID-19 registry and identified 1,115 encounters, 182 with ≥1 AGP. Overall, COVID-19 incidence among EMS personnel was 0.57 infections/10,000 person-days. Incidence per 10,000 person-days did not differ whether or not infection was attributed to a COVID-19 patient encounter (0.28 vs. 0.59; p>0.05). The 1 case attributed to a COVID-19 patient encounter occurred within an at-risk period and involved an AGP. We observed a very low risk for COVID-19 infection attributable to patient encounters among EMS first responders, supporting clinical strategies that maintain established practices for treating patients in emergency conditions.

Dynamic circumstances, time sensitivity, limited information about widely variable scenes encountered, and heterogeneous patient characteristics make emergency medical service (EMS) responses inherently challenging. The global coronavirus disease (COVID-19) pandemic, caused by severe acute respiratory syndrome coronavirus 2 (SARS-CoV-2), has now forced EMS providers to also consider how best to manage their own potential exposure, particularly when a patient’s infection status is unknown (*1*,*2*).

During outbreaks of severe acute respiratory syndrome in 2003 and Middle East respiratory syndrome in 2012, many healthcare workers became infected while caring for patients (*3*–*5*). There is an evolving understanding of the risk of patients transmitting COVID-19 to healthcare workers, but less is known about transmitting it to emergency medical first responders or about the specific etiology of infection (*6*–*10*).

Respiratory exposure is the primary mode of COVID-19 transmission (*11*,*12*). Clinical guidelines have evolved to mitigate risk for transmission, especially through aerosolizing procedures used for cardiopulmonary resuscitation (CPR) or airway management. A better understanding of the risks related to patient care itself could further inform clinical practice approaches, therapeutic choices, and personal protective equipment (PPE) strategies in an effort to balance risks and benefits for providers and patients while striving to maintain best practices for patient care (*4*,*12*,*13*). Therefore, we investigated the risk for COVID-19 transmission from patient to provider and how use of aerosol-generating procedures (AGP) during the encounter might affect risk levels.

## Methods

### Study Design, Population, and Setting

We conducted a retrospective cohort study to evaluate the risk for COVID-19 infection among EMS providers caring for patients in King County, Washington, USA, during February 16–July 31, 2020. When determining risk for COVID-19, we considered all EMS provider-patient encounters and individual EMS providers involved in those encounters. The investigation was designed and reported with consideration of the Strengthening the Reporting of Observational Studies in Epidemiology (STROBE) reporting guidelines (*14*) and approved by the University of Washington and Seattle and King County Public Health and University of Washington public health review boards.

King County is a large metropolitan region encompassing the city of Seattle and covering ≈2,300 square miles with ≈2.3 million residents living in urban, suburban, and rural areas. The EMS system is 2-tiered, the first tier comprising 27 firefighter and emergency medical technician departments and the second tier 5 paramedic departments serving multiple emergency medical technician departments for responding to more serious medical emergencies. EMS teams of 2–7 providers respond to calls based on dispatcher-determined acuity. In general, fire department or private basic life support ambulance units transport medically stable patients to hospitals and advanced life support paramedic units transport patients needing more acute care.

### EMS COVID-19 Protocols

Seattle and King County EMS management developed protocols for screening and care of patients at risk for having COVID-19 (*15*). EMS PPE protocols include wearing a mask, eye protection, gloves, and a gown. Surgical masks were considered sufficient for treating patients not requiring AGP, but an N95 respirator was required when patients underwent AGPs. HEPA (high efficiency particulate air) filters were added to ventilation bags. Otherwise, clinical protocols did not change in response to the pandemic. For example, the EMS system continued to support the use of endotracheal intubation and manual CPR to treat out-of-hospital cardiac arrest (*13*).

### Data Sources, Linkages, and Abstraction

The Seattle and King County EMS Division of Public Health maintains an encounter-level electronic health record of each EMS response using software from ESO Solutions Inc. (https://www.eso.com). The EMS record for each incident contains information about patient and EMS provider identities, chief complaints, signs and symptoms, EMS care, and PPE use by providers. The state of Washington Disease Reporting System (WDRS) contains names, dates of birth, test dates, and results for all persons who have been tested for SARS-CoV-2 within the state. Seattle and King County Public Health administers the EMS system, enabling identification of EMS encounters with patients who have COVID-19 (*15*). To obtain patient COVID-19 status, we linked WDRS with EMS electronic health records using a multistep algorithm including the patient’s first and last names and date of birth; identification through this linkage was followed by human confirmation of the potential link.

In addition to the linking process for COVID-19 status, we determined the health-related vital status of patients with COVID-19 by linking those patients with Washington State Department of Health vital records available through December 1, 2020, All study information for COVID-19 patient encounters was abstracted into a secure Research Electronic Data Capture (REDCap, https://www.project-redcap.org) platform by using a uniform data abstraction form supported by a data dictionary (*16*). The abstract recorded a review of the narrative and discrete data fields from the dispatch and EMS records.

### Exposure and Data Definitions

#### COVID-19 Patient Classification

A provider was considered to have encountered a patient with COVID-19 if the patient had a positive SARS-CoV-2 swab sample result determined by using real-time reverse transcription PCR (rRT-PCR) ≤10 d before or ≤3 d after an EMS encounter, on the basis of data from the linked EMS and WDRS records. We chose ≤10 d as a criterion on the basis of the 10-day infectious window after onset of symptoms. We used ≤3 d as a criterion after the EMS encounter recognizing that not all patients had been tested upon hospital arrival, especially in the first few months of the pandemic. In addition, a minority of patients were not transported by EMS and had subsequent follow-up for testing even though the EMS encounter appeared to be for illness consistent with COVID-19 (*2*).

#### AGP Definition and Classification

For this study, we classified endotracheal intubation, supraglottic airway insertion, bag-valve-mask (BVM) ventilation, continuous positive airway pressure nonrebreather mask oxygen, and nebulizer medication therapy as AGPs (*4*). Although the standards for AGP are not fully defined, nonrebreather masks routinely involve using higher-flow oxygen (15 L/min) and require applying and manipulating face masks, which may increase transmission risk (*4*,*17*,*18*). We did not classify use of low-flow nasal cannula oxygen as an AGP. In an EMS patient-encounter setting, CPR always involves both chest compressions and BVM ventilation, which constitutes an AGP. We identified AGP procedure usage from the EMS records by searching electronic text records for key phrases in the narratives or discrete electronic data elements that recorded AGP procedures. We evaluated the accuracy of this method to identify AGP by manually reviewing records of all EMS encounters with COVID-19 patients.

### Classifying EMS Provider Person-Days at Risk

For each day of the study period, each EMS provider’s day was classified into 1 of 4 mutually exclusive cohorts based on the time interval after COVID-19 patient encounters, if any, and whether or not AGPs were used. Person-days were classified into cohort 1 for COVID-19 patient encounters that involved ≥1 AGPs during the 2–14 d incubation period, cohort 2 for COVID-19 patient encounters that did not involve AGPs during the 2–14 d incubation period, cohort 3 for COVID-19 patient encounters before or after the 2–14 d incubation period, or cohort 4 if the provider had no COVID-19 patient encounters during the study period. Individual EMS providers could contribute discrete person-days to different cohorts, except for cohort 4.

**Figure 1:**
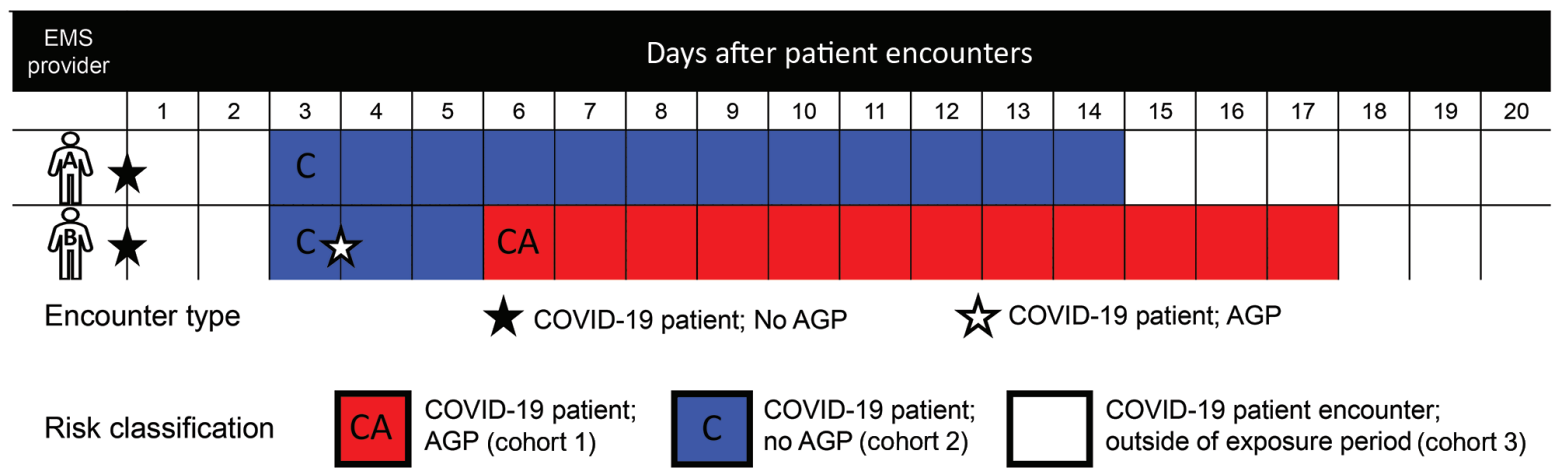
Examples of classification of EMS provider person-days at risk within 2–14 d after COVID-19 patient encounters, King County, Washington, February 16–July 31, 2020. The boxes correspond to the number of person-days an emergency medical services provider contributes to each mutually exclusive risk group. The first row (provider A) demonstrates a COVID-19 patient encounter without an AGP. The provider is classified at risk for COVID-19 transmission because of a patient treated without AGP within 2–14 d after encounter. After the incubation window ends, the EMS provider transitions back to person-days classification of COVID-19 patient outside the incubation period (cohort 3). The second row (provider B) demonstrates classification of person-days from COVID-19 patient without AGP and then with AGP. Person-days transitions from COVID-19 patient encounter without AGP (cohort 2) to patient encounter with AGP (cohort 1). The example illustrates the classification hierarchy that classified the patient into the AGP incubation period when a provider had overlap of person-days following distinct encounters caring for COVID-19 patients without an AGP and then with an AGP. After the incubation window, the EMS provider will transition back to person-days classification of COVID-19 patient outside the incubation period (group 3). AGP, aerosol-generating procedure; COVID-19, coronavirus disease; EMS, emergency medical service.

We considered EMS providers at risk for transmission from a patient for 2–14 d after an encounter with a COVID-19 patient (Figure 1), because the biology of transmission and illness indicates that the COVID-19 incubation period is 2–14 d (*19*). If an EMS provider tested positive for SARS-CoV-2 in the 2–14 d incubation period after treating a COVID-19–positive patient, the infection was attributed to the encounter. For classification, once an EMS provider completed the 14 d incubation period without SARS-CoV-2 infection, the provider’s person-days for subsequent days would transition from cohort 1 or 2 to cohort 3 until the provider was involved with another patient with COVID-19.

For days when a provider had multiple COVID-19 patient encounters and ≥1 involved an AGP, the provider’s person-hours for that day were classified into cohort 1, given that AGP use is considered to possess greater intrinsic transmission risk. EMS providers could be diagnosed with COVID-19 on a person-day in any of the 4 cohorts. After a provider’s first rRT-PCR–positive SARS-CoV-2 swab result, they were censored from the analysis and did not contribute additional person-days to any cohort. SARS-CoV-2 reinfection was not diagnosed in any provider.

### Outcome Measures

We used COVID-19 infections among EMS providers as determined from the WDRS registry during February 15–August 14, 2020, as the primary outcome measure. We extended the period for assessing COVID-19 to August 14, two weeks beyond the final day for recording person-days, to ensure we captured infections identified ≥14 d after COVID-19 patient encounters within the study period.

As part of COVID-19 surveillance, EMS implemented a screening process for potential COVID-19 illness among EMS personnel at the outset of each shift comprising a temperature check and observation for symptoms of medical illness. EMS personnel were guided by a return-to-work algorithm that recommended COVID-19 rRT-PCR testing for any acute illness acquired on or off duty in an effort to limit the risk of provider-to-provider transmission and maintain workplace safety (*15*).

### Analysis

We performed descriptive analyses at the encounter, patient, and EMS provider levels. We stratified provider encounters and classified person-days according to patient COVID-19 status and whether or not treatment included >1 AGPs. EMS providers were censored from the study on the date they were diagnosed with COVID-19 or at the end of the follow-up period (August 15, 2020) if never diagnosed with COVID-19. We then calculated the incidence of COVID-19 infection among EMS providers on the basis of person-days at risk from COVID-19 patient encounters. We calculated the incidence rate ratio using the collective person-days from cohort 3, the cohort including person-days before or after the 2–14 d incubation period of a COVID-19 patient encounter, as the referent group because this approach enabled providers to serve as their own controls when evaluating the risk attributable to COVID-19 patient encounters. In a post hoc analysis, we combined the person-days from cohorts 1 and 2 to evaluate the overall COVID-19 incidence among EMS providers attributed to a COVID-19 patient encounter regardless of AGP use.

## Results

### Encounters with COVID-19 Patients

**Figure 2:**
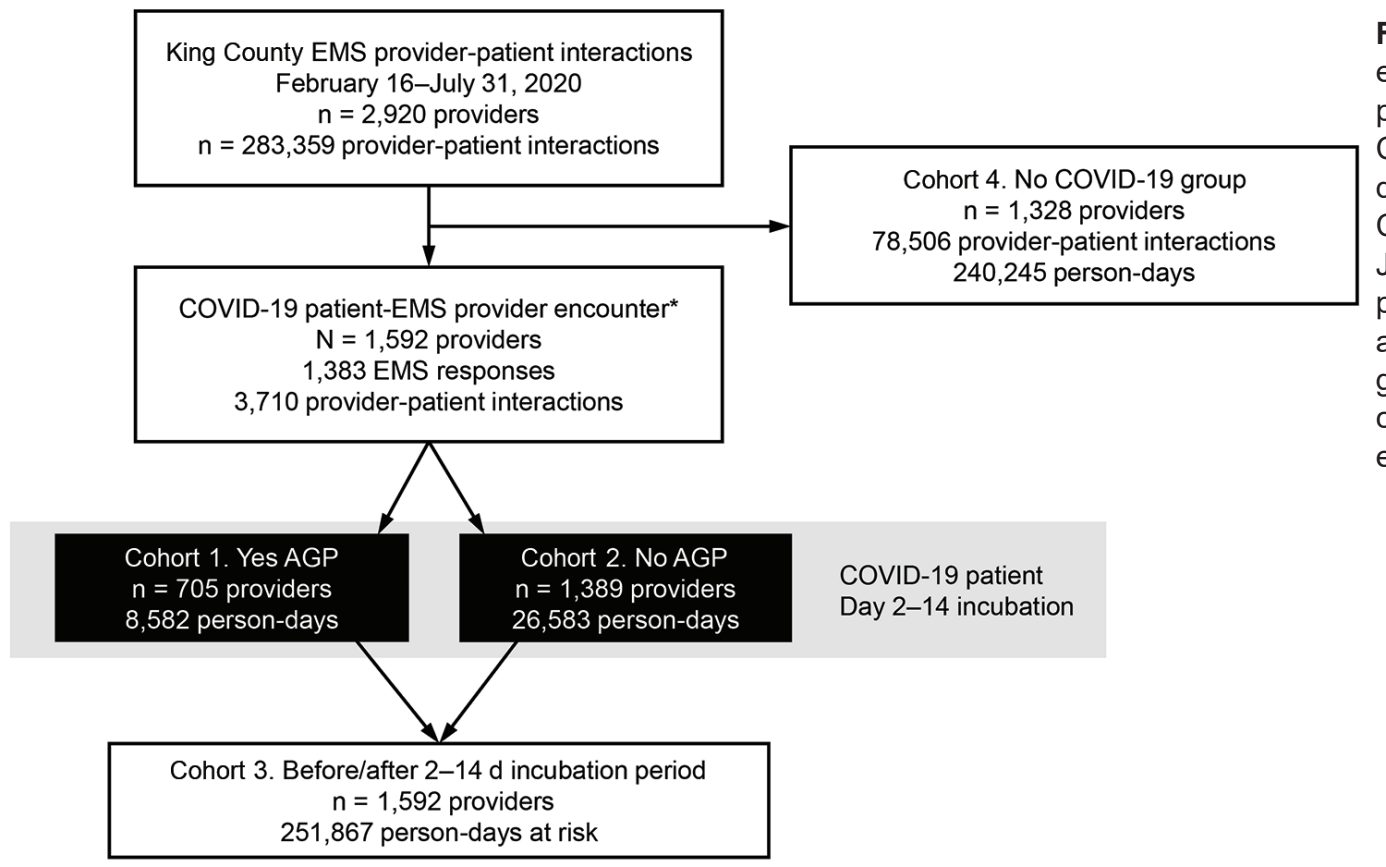
Flow diagram of emergency medical service provider encounters with COVID-19 patients and person-days at risk for transmission, King County, Washington, February 16–July 31, 2020. Individual provider’s person-days may transition among cohorts 1–3. AGP, aerosol generating procedure; COVID-19, coronavirus disease; EMS, emergency medical services.

During the February 16–July 31, 2020, study period, 1,592 different EMS providers cared for 946 unique COVID-19 patients as part of 1,115 EMS responses, resulting in 3,710 provider-patient COVID-19 encounters. Over that period, 1,328 EMS providers did not care for any patients in whom COVID-19 had been diagnosed. Cohorts 1–3 encompassed a total of 287,032 person-days in which there were COVID-19 patient encounters, and cohort 4 encompassed a total of 240,245 person-days in which there were no COVID-19 patient encounters (Figure 2). Among the 1,592 EMS providers with ≥1 COVID-19 patient encounter, 655 (41%) had 1 encounter, 417 (26%) had 2, and 520 (33%) had ≥3.

We recorded details from the 1,115 encounters involving ≥1 provider and ≥1 COVID-19 patient, overall and stratified by AGP status (Table 1). An AGP was performed in 182 (16%) patient encounters involving 787 EMS providers (567 different providers). Overall, half of the EMS responses were for female patients; the average patient age was 68 years. About half of EMS responses were to private residences and 41% to long-term care or assisted living facilities. Responders reported ≥1 clinical signs of shortness of breath (42%), cough (36%), or fever (42%) in 67% of patients. In the cohort of provider person-days when using AGPs (cohort 1) compared with the cohort of person-days when not using AGPs (cohort 2), patient encounters were more often characterized by tachypnea (63% vs. 28%), hypoxemia (70% vs. 18%), abnormal heart rate (48% vs. 38%), systolic blood pressure <90 mm Hg (17% vs. 4%), and Glasgow coma scale ≤12 (25% vs. 6%). The most common EMS provider-recorded impression of patient illness overall among the 1,115 responses was respiratory distress (n = 417, 37%), 24% (n = 101) of those among patients needing AGPs and 76% (n = 316) among patients not needing AGPs. Twenty-two patients had out-of-hospital cardiac arrests, comprising 12.1% of the provider person-days in cohort 1 (Table 1). The most common AGP provided was nonrebreather mask oxygen (n = 139) (Table 2). Other common AGPs included BVM ventilation (n = 42) and endotracheal intubation (n = 29). Among patient encounters grouped in the first cohort, 44 (24%) involved >1 AGP during a single encounter, most often nonrebreather oxygen followed by BVM ventilation, then intubation. Overall, 34% of COVID-19 patients, 57% of those receiving AGPs and 29% of those not receiving AGPs, died during follow-up from the time of encounter through December 31, 2020.

**Table 1:** Encounter characteristics by aerosol generating procedure status among COVID-19 patients, King County, Washington, February 16–July 31, 2020*

Characteristic	All encounters	AGP encounters	Non–AGP encounters
Unique encounters	1,115 (100.0)	182 (16.3)	933 (83.7)
Patient age, mean (SD)	68.1 (19.8)	69.4 (18.4)	67.8 (20.1)
Sex			
M	563 (50.5)	100 (54.9)	463 (49.6)
F	552 (49.5)	82 (45.1)	470 (50.4)
Location			
Home	529 (47.4)	78 (42.9)	451 (48.3)
Long-term care	458 (41.1)	87 (47.8)	371 (39.8)
Public outdoors	50 (4.5)	3 (1.6)	47 (5.0)
Medical clinic or office	38 (3.4)	11 (6.0)	27 (2.9)
Public indoors	21 (1.9)	1 (0.5)	20 (2.1)
Homeless shelter	19 (1.7)	2 (1.1)	17 (1.8)
Other	Unknown	Unknown	Unknown
Documented signs and symptoms			
Fever	467 (41.9)	74 (40.7)	393 (42.1)
Cough	401 (36.0)	65 (35.7)	336 (36.0)
Shortness of breath	472 (42.3)	133 (73.1)	339 (36.3)
Fever/cough/shortness of breath	751 (67.4)	147 (80.8)	604 (64.7)
Sore throat/nasal congestion	79 (7.1)	8 (4.4)	71 (7.6)
GI symptoms	160 (14.3)	23 (12.6)	137 (14.7)
Body aches	175 (15.7)	27 (14.8)	148 (15.9)
Altered mental status	188 (16.9)	39 (21.4)	149 (16.0)
Fatigue/weakness	354 (31.7)	38 (20.9)	316 (33.9)
Headache	37 (3.3)	6 (3.3)	31 (3.3)
Chest pain	75 (6.7)	11 (6.0)	64 (6.9)
Vital signs			
Any abnormal vital sign	936 (83.9)	179 (98.4)	757 (81.1)
Heart rate ≥100 bpm	438 (39.3)	87 (47.8)	351 (37.6)
Temperature ≥38°C	573 (51.4)	94 (51.6)	479 (51.3)
Respirations ≥24 breaths/min	378 (33.9)	114 (62.6)	264 (28.3)
Oxygen saturation ≤90 SpO_2_	292 (26.2)	127 (69.8)	165 (17.7)
Systolic blood pressure ≤90 mm Hg	69 (6.2)	30 (16.5)	39 (4.2)
Glasgow coma scale <12	97 (8.7)	46 (25.3)	51 (5.5)
Glasgow coma scale 13–14	58 (5.2)	20 (11.0)	38 (4.1)
Glasgow coma scale = 15	534 (47.9)	76 (41.8)	458 (49.1)
Patient with cardiac arrest	22 (2.0)	22 (12.1)	Unknown
Initial EMS response type			
Respiratory	417 (37.4)	101 (55.5)	316 (33.9)
Fatigue/weakness/malaise	157 (14.1)	8 (4.4)	149 (16.0)
Infection	128 (11.5)	13 (7.1)	115 (12.3)
Behavioral/psychological/intoxication	114 (10.2)	19 (10.4)	95 (10.2)
Other medical	72 (6.5)	2 (1.1)	70 (7.5)
Cardiovascular	64 (5.7)	29 (15.9)	35 (3.8)
Trauma	64 (5.7)	5 (2.7)	59 (6.3)
Abdominal/GU/endocrine	60 (5.4)	3 (1.6)	57 (6.1)
Neurological	39 (3.5)	2 (1.1)	37 (4.0)

*Values are no. (%) except as indicated. AGP, aerosol generating procedure; COVID-19, coronavirus disease; EMS, emergency medical service; GI, gastrointestinal; GU, genitourinary.

**Table 2:** Patient outcome emergency medical service care and by aerosol generating procedure status among COVID-19 patients, King County, Washington, February 16–July 31, 2020*

Characteristic	All encounters	AGP encounters	Non–AGP encounters
Unique EMS providers	1,592	567	1,025
EMS encounters	1,115	182	933
ALS unit dispatched	171 (15.3)	98 (53.8)	73 (7.8)
EMS suspicion of COVID-19	715 (64.1)	132 (72.5)	583 (62.5)
Low-flow oxygen	188 (16.9)	34 (18.7)	154 (16.5)
AGP types			
Nonrebreather	139 (12.5)	139 (76.4)	NA
Simple face mask	5 (0.4)	5 (2.7)	NA
Medication therapy	13 (1.2)	13 (7.1)	NA
Metered dose inhaler	4 (0.4)	4 (2.2)	NA
Nebulizer	9 (0.8)	9 (4.9)	NA
NiPPV	48 (4.3)	48 (26.4)	NA
CPAP	6 (0.5)	6 (3.3)	NA
Bag-valve-mask ventilation	42 (3.8)	42 (23.1)	NA
Suction	4 (0.4)	4 (2.2)	NA
Advanced airways	32 (2.9)	32 (17.6)	NA
Supraglottic airway	3 (0.3)	3 (1.6)	NA
Endotracheal intubation	29 (2.6)	29 (15.9)	NA
AGP frequency per encounter			
0	933 (83.7)	0	933 (100)
1	138 (12.4)	138 (75.8)	NA
≥2	44 (3.9)	44 (24.2)	NA
Disposition			
Not transported	245 (22.0)	21 (11.5)	224 (24.0)
BLS transport	759 (68.1)	108 (59.3)	651 (69.8)
ALS transport	86 (7.7)	53 (29.1)	33 (3.5)
Private vehicle	17 (1.5)	0	17 (1.8)
Air ambulance	1 (0.1)	1 (0.5)	0
Patient mortality as of 2020 Dec 1	373 (33.5)	103 (56.6)	270 (28.9)

*Values are no. (%). AGP, aerosol generating procedure; ALS, advanced life support; COVID-19, coronavirus disease; CPAP, continuous positive airway pressure; EMS, emergency medical service; NA, not applicable; NiPPV, noninvasive positive-pressure ventilation.

### EMS Provider Risk

The 2,920 EMS providers followed over the 181-day study period produced 525,154 person-days at risk: 8,582 person-days from 705 providers treating COVID-19 patients using AGP within the incubation period (cohort 1); 26,583 person-days from 1,389 providers treating COVID-19 patients without AGP within the incubation period (cohort 2); 252,867 person-days from 1,592 providers treating COVID-19 patients outside the incubation period (cohort 3); and 240,245 person-days from 1,328 providers who never treated a COVID-19 patient during the study period (cohort 4). Thirty EMS providers had positive rRT-PCR COVID-19 test results (Table 3). The median interval between COVID-19 patient encounter and EMS provider positive rRT-PCR test was 73 days (IQR 30–105 days). Only 1 infection occurred within the 2–14-d window after an encounter with a COVID-19 patient; during that period, the provider encountered >1 COVID-19 patient with ≥1 involving AGP use, so transmission was attributed to a patient encounter in which an AGP was provided. An additional 18 EMS providers cared for COVID-19 patients and acquired COVID-19. However, their COVID-19–positive tests were outside the 2–14-d incubation period after caring for a patient with COVID-19. Eleven EMS providers who never cared for a patient with COVID-19 tested positive for COVID-19.

**Table 3:** Incidence of COVID-19 among EMS providers by COVID-19 patient encounter and AGP status cohort, King County, Washington, February 16–July 31, 2020*

Cohort	COVID-19 patient encounter	2–14 d exposure window	AGP status	EMS provider COVID-19 infection	Person-days at risk	Incidence/10,000 person-days (95% CI)	IRR (95% CI)
1	Yes	Yes	Yes	1	8,582	1.17 (0.03–6.49)	1.64 (0.22–12.26)
2	Yes	Yes	No	0	26,583	0 (0.0–1.39)	0 (0.0–1.50)
3	Yes	No	NA	18	252,867	0.71 (0.42–1.13)	Referent
4	Never	NA	NA	11	240,245	0.46 (0.23–0.82)	0.64 (0.30–1.36)
Post hoc							
1 and 2	Yes	Yes	Y/N	1	35,165	0.28 (0.01–1.58)	0.40 (0.05–2.99)

*AGP, aerosol generating procedure; COVID-19, coronavirus disease; EMS, emergency medical service; IRR, incidence rate ratio; NA, not available

Overall, the incidence of rRT-PCR positive tests among EMS providers was 0.57/10,000 person-days (30 positive tests in 525,154 person-days). The relative risk associated with COVID-19 patient encounters, with or without AGP use, did not differ compared with those without any COVID-19 patient encounters (Table 3). Finally, we found no difference in incidence between aggregated person-days attributed to COVID-19 patient encounters, 0.28/10,000 person-days (1 positive test in 35,165 person-days), and person-days not attributed to COVID-19 patient encounters, 0.59/10,000 person-days (29 positive tests in 489,989 person-days; p>0.05).

## Discussion

In this observational study of a populous US metropolitan region, encounters with patients with COVID-19 accounted for 1% of all 911 EMS responses, involving nearly 1,200 unique COVID-19 patients and several thousand patient-provider encounters during the study period. Approximately 16% of these COVID-19 patient encounters involved treatment with AGPs, typically for patients with more severe illness based on field assessment and underscored by subsequent all-cause death rates. However, risk for the first responder workforce primarily originated from nonpatient sources; 29 of 30 COVID-19 illnesses among EMS providers were not directly attributed to COVID-19 patient encounters. Collectively, the results suggest that PPE provides protection against acquiring COVID-19 during prehospital emergency patient care, which supports maintenance of established practices.

 Although the results indicate that risk of transmission from patients is low, the findings also highlight potential for concern. COVID-19 patients comprised only 1% of EMS responses, but that small fraction translated to thousands of calls involving ≈55% of the region’s first-responder workforce over the 6 months of our investigation. One third of COVID-19 patients did not display any common symptoms, such as fever, coughing, or shortness of breath (*2*), and about one sixth of all COVID-19 patient encounters involved a prehospital AGP. Collectively, the involvement of such a large proportion of the first responder workforce, the heterogeneous nature of patient characteristics, and the time-pressured need among some patients for AGP intervention could pose major COVID-19 risk to public safety personnel and infrastructure. This reality needs to be considered not only with regard to COVID-19 but also to future infectious disease risks, including as part of pandemics.

In our study, however, we found a low overall risk of EMS provider infection from patient care; COVID-19 occurred in a single provider in 1 of 3,710 provider-patient encounters, representing an incidence of 0.28 cases/10,000 person-days at risk. The low incidence occurred under circumstances in which ample PPEs were available for EMS providers and public health management provided active oversight to support guideline-directed PPE field practices (*15**,**20*). The low infection rate attributed to patient care covered 182 COVID-19 patient encounters when AGPs were used, including the spectrum of high-flow oxygen, advanced airway maneuvers, and attempted resuscitation. Although data from larger numbers of patient encounters with use of different AGPs could perhaps help researchers refine the overall estimate and potentially determine treatment-specific risk, the overarching inference is that PPE provides excellent protection under these prehospital circumstances. The findings should reassure first responders that emergency care in general and specifically when using AGPs can be delivered safely to treat patients as long as PPE are properly deployed and that, in general, EMS personnel and management should not change evidence-based practice solely to mitigate transmission risk.

Our results also highlight the realities of the COVID-19 pandemic. Sources of infectious risk for EMS personnel are not confined to patients. We observed that the large majority of COVID-19 illness was a consequence of encounters not with patients but in the community or occupational settings. These findings support efforts to screen workplaces for provider symptoms or initiate point-of-care provider testing to limit on-the-job exposure as well as to practice guideline-directed social distancing, masking, and hygiene recommendations outlined for the general public, acknowledging that vaccination may affect these directives (*21*).

The study leveraged linking electronic records to establish EMS provider–COVID-19 patient encounters, but the data platforms or linkages may not have been comprehensive. Specifically, the registry of persons positive for COVID-19 requires a test, so we could have underestimated the risk attributable to encounters with untested patients. However, in the study methodology we attributed a priori an EMS provider’s COVID-19 infection to a patient encounter if it occurred within 2–14 days after the encounter, even though the transmission could have originated from another source. Conversely, this design approach could have overestimated the risk attributable to the COVID-19 patient encounter because the study did not specifically evaluate nonpatient sources of SARS-CoV-2 provider infection (including transmission among co-workers). We defined AGP on the basis of prior research. Although the results from our study were clinically encouraging, the small number of patient encounters limited our ability to compare encounters with patients by whether AGPs were used or not and by the different types of AGPs.

This active evaluation in the context of the region’s EMS operational structure and the profile of experienced EMS providers may influence the generalizability of the results. For example, each year the Seattle and King County EMS system’s providers are required to review and be tested on the topic of occupational infectious diseases. As part of the standard approach to patient care before the pandemic, EMS personnel routinely wore gloves and eyewear and were regularly fit-tested for N95 masks, so PPE use was to some extent already common practice at the outset of the pandemic. Moreover, the EMS system has been able to ensure PPE supply to achieve guideline-directed practices during the pandemic. These study-specific characteristics should be considered in balance with the study’s broader strengths: innovative linking across EMS records and with the SARS-CoV-2 test registry, reviewing and classifying AGP status for each COVID-19 patient encounter, and undertaking a population-based regional evaluation.

In summary, we observed a very low overall risk for COVID-19 infection among the EMS first-responder workforce attributed to COVID-19 patient encounters, although the small number of EMS provider infections prevented definitive inference regarding AGP-specific risk. These findings support clinical strategies that maintain established, evidence-based practices for emergency conditions. Future efforts should continue to evaluate care settings, patient medical characteristics, provider behaviors, specific treatments, and systemwide PPE availability and status to establish risk and refine prevention practices.
